# DePicT Melanoma Deep-CLASS: a deep convolutional neural networks approach to classify skin lesion images

**DOI:** 10.1186/s12859-020-3351-y

**Published:** 2020-03-11

**Authors:** Sara Nasiri, Julien Helsper, Matthias Jung, Madjid Fathi

**Affiliations:** Department of Electrical Engineering and Computer Science, University of Siegen, Hölderlinstr. 3, Siegen, Germany

**Keywords:** Deep learning, Early detection, Skin cancer, Melanoma, Classification, Case-based reasoning, Information retrieval

## Abstract

**Background:**

Melanoma results in the vast majority of skin cancer deaths during the last decades, even though this disease accounts for only one percent of all skin cancers’ instances. The survival rates of melanoma from early to terminal stages is more than fifty percent. Therefore, having the right information at the right time by early detection with monitoring skin lesions to find potential problems is essential to surviving this type of cancer.

**Results:**

An approach to classify skin lesions using deep learning for early detection of melanoma in a case-based reasoning (CBR) system is proposed. This approach has been employed for retrieving new input images from the case base of the proposed system DePicT Melanoma Deep-CLASS to support users with more accurate recommendations relevant to their requested problem (e.g., image of affected area). The efficiency of our system has been verified by utilizing the ISIC Archive dataset in analysis of skin lesion classification as a benign and malignant melanoma. The kernel of DePicT Melanoma Deep-CLASS is built upon a convolutional neural network (CNN) composed of sixteen layers (excluding input and ouput layers), which can be recursively trained and learned. Our approach depicts an improved performance and accuracy in testing on the ISIC Archive dataset.

**Conclusions:**

Our methodology derived from a deep CNN, generates case representations for our case base to use in the retrieval process. Integration of this approach to DePicT Melanoma CLASS, significantly improving the efficiency of its image classification and the quality of the recommendation part of the system. The proposed method has been tested and validated on 1796 dermoscopy images. Analyzed results indicate that it is efficient on malignancy detection.

## Background

Although case-based reasoning (CBR) has been applied in a number of medical systems, only a few systems have been developed for melanoma. The estimated 5-year survival rate for patients whose melanoma is detected early is about 99% [1] in the USA and around 93% [2] in Germany. The survival rate falls to 63% when the disease reaches the lymph nodes and 20% when the disease metastasizes to distant organs [1]; therefore, having the right information at the right time by developing decision support systems is essential and has become a major area of research in this field. The best path to early detection is recognizing new or changing skin growths, especially those that appear different from other moles [3]. Even after treatment, it is very important for patients to keep up on their medical history and records. The national comprehensive cancer network (NCCN) which is an alliance of 28 cancer centers in the United States, creates helpful reports and resources to serve as guidelines for informing patients and other stakeholders about cancer [4]. In this paper, we propose a hybrid CBR system and evaluate its performance on the skin lesions classification (benign and malignant). In the proposed system, deep neural networks are used as an image classifier in the context of CBR methodology and in the retrieval process. The case base of our proposed system contains melanoma images and skin cancer information as a case description and recommendation and the structure of cases includes image features, identified keywords, and a word association profile, which is explained in the next section. The case-base updates and learns over the time by new images. Image-based classification enables the system to give reasons for selecting matched cases and solutions to the problem description. Therefore this hybrid system has the advantages of deep learning (DL) and CBR and benefits from both. DL helps researchers absolutely to treat and detect diseases by analyzing medical data (e.g., medical images). One of the representative models among the various deep-learning models is a convolutional neural network (CNN). This classification of skin cancer by CNN as an image-based classification is comparable to the dermatologists’ detection [5].

Besides most groups trust in CNNs in recent researches, some are still using other methods. Mustafa and Kimura followed an approach for melanoma classification based on manually developing and selecting features [6]. Lesions of interest are segmented using the GrabCut algorithm. Shape, color, and geometry are the selected feature categories. A support vector machine (SVM) with Gaussian radial basis kernel (SVM-RBF) is used to differentiate between cancerous “malignant” or non-cancerous mole “benign” lesions. Zakeri and Soukhtesaraie developed a support system based on a log-linearized Gaussian mixture neural network (LLGMNN) [7]. First steps involve the removing of artifacts, then Otsu thresholding method helps to detect lesions. Features, including border, shape color and texture based, are selected and extraction of the most promissing is performed. Two different validated LLGMNN designs serve as classifier for the system. One detects melanoma from non-melanoma lesions and the other differentiates between melanoma, dysplastic, and benign lesions. Since basal cell carcinoma are the most common type of skin cancer systems for their detection are necessary as well. E. Vander Putten et al. developed a system for early detection [8]. The system follows three major steps. At first the input image is preprocessed. Afterwards the minimum area of the image is extracted with a black and white lesion map. Finally a ’very’ deep residual network is used for classification giving a probability value as output. Cherepkova and Hardeberg [9] focused on the benefits for melanoma skin cancer classifier when the trainings data is augmented with different methodologies beforehand. Image enhancement and color correction methods are compared and evaluated. Our previous works included training SVMs and k-nearest neighbors (K-NNs) to differentiate between melanoma and other types of skin lesions [10]. Several features were developed and the 12 best performing got selected. First results for testing the classification of our system with a CNN based approach were also introduced in our previous work [11].

CNNs have emerged to be one of the maior techniques for image classification in the last few years. A large number of improvements have been made. One of the problems of convolutional neural networks is overfitting. Srivastava et al. introduced dropout as a technique to avoid this problem [12]. Dropout randomly disabels neurons with a probability p which avoids co-adapting. Dahl et al. pointed out that dropout increases the time needed for training. They suggested rectified linear units as replacement for sigmoid units and prove that neural networks are faster to train this way [13]. Also Dahl et al. revealed that using rectified linear units over sigmoid units can improve the networks performance.

There are several techniques in data augmentation which improve the variety of data for training. In [14], Wang and Perez compared different solutions for the problem of data augmentation in image classification. Paulin et al. described different data augmentation techniques and their benefits [15]. How to use techniques of data augmentation is explained in [16] by B. Raj.

’AlexNet’ developed by Krizhevsky et al., is a well known net for classifying objects in several problems [17]. Compared to other nets in the past AlexNet excelled in exploiting the benefits of GPU usage and the advantages of the dropout layer. D. Scherer et al. evaluated different pooling methods in regards of neural network performance [18]. According to the paper overlapping pooling windows show no significant improvement compared to non-overlapping pooling windows. Furthermore max pooling outperforms other pooling methods in most cases. Simonyan and Zisserman discussed about the different CNNs architectures and the importance of its depth [19]. The 16 and 19 weight layers architectures are well known under the names Visual Geometry Group (VGG), VGG16 and VGG19 respectively. Those architectures feature only 3x3 convolutional layers, 5 max-pooling layers and 3 fully-connected layers. ’GoogLeNet’ by Szegedy et al. is a 22 layer deep net architecture also known as Inception v1 [20]. It features a 1x1 convolutional neural network in the middel and uses global average pooling instead of fully connected layer at the end.

Yu et al. deal with the detection of Acral melanoma, the most common type of melanoma in Asians, in an early stage[21]. A CNN is proposed and applied to dermoscopy images of acral melanoma and benign nevi located on hands and feet. In the resulting problem they received a 83.51% accuracy.

As basis for our research serves the data from the ’ISIC-Archive’ [22] dataset. Several groups have participated in the so called ’ISIC 2017’ challenge in order to create well performing classifier. Three classes, malignant melanoma, nevocellular nevus, and seborrheic keratosis were subjects of this challenge. Two separate linear classifiers, malignant melanoma versus all and seborrheic keratosis versus all, had to be created. Matsunaga et al. first performed normalization of luminanz and color [23]. Transformation including rotation, translation, scaling and flipping is performed. A modified version of the 50-layer ’ResNet’ architecture [24] is used for classification. González-Díaz [25] used a ’Lesion Segmentation Network’ for each clinical case in the first step. Segmented results are further processed in a ’Data Augmentation Module’ which generates more images. A Structure ’Segmentation Network’ segmented relevant local and global structures. Finally the augmented data is classified by a ’Diagnosis Network’. Mengalo et al. followed an approach training several models separately and combining the results [26]. Models for training are based on ’ResNet’ [24] architecture and ’Inception-v4’ [27] architecture. For training step, different subsets in different sizes are used and results are combined using SVM. Li and Shen [28] generated two datasets with more images via rotation and rescaling. They trained two fully convolutional residual networks, called FCRN-88. Afterwards bilinear interpolation is performed and results are summed up. For classification they compared one distance map with three possibility maps to obtain one index for each category of skin lesion.

The structure of our paper is as follows: “Background” section, briefly described the background in terms of related works. “Methods” section focuses on the methodology by starting to explain the preliminary system called DePicT Melanoma CLASS. The proposed system of current research is followed in “Methods” section by a description of image processing and classification with deep neural networks. The “Datasets and tools” section explains the tools and dataset which we have used for the implementation of our system. “Results and discussion” section discusses the results and evaluation. Finally, “Conclusions” section concludes the paper.

## Methods

### DePicT Melanoma CLASS

**Fig. 1:**
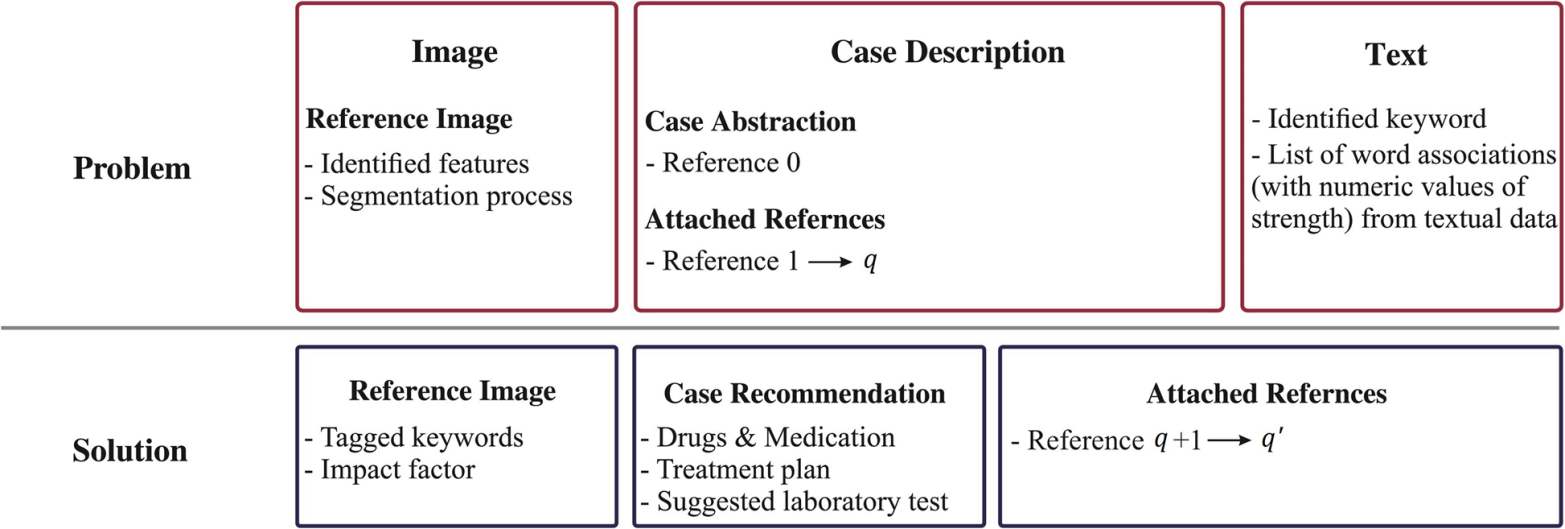
Case representation. Case structure of DePicT Melanoma CLASS [10]

The first clinical signs of melanoma as lesions denotes the affected area and corresponding spots on the skin. Various skin lesions classification systems have been developed using SVMs and k-NNs like interactive object recognition methodologies to perform border segmentation [29], extract global and local features and apply Otsu’s adaptive thresholding method [30]. Sumithra et al. utilized SVM and k-NN for skin cancer classification based on region-growing segmentation with results of 46% and 34% (f-measure), respectively [31]. In the previous study, DePicT CLASS [32] was used to retrieve the textual components of requested problems and classify melanoma images using region growing methods based on SVM and k-NN to support patients and health providers in managing the disease [10]. The case base of DePicT Melanoma CLASS is built based on the AJCC (American Joint Committee on Cancer: Melanoma of the Skin Staging) staging, melanoma skin cancer information data base [https://www.cancer.org/cancer/melanoma-skin-cancer.html] and melanoma images from the ISIC archive dataset [22]. Each case has a word association profile for main keywords extracted from melanoma textbooks and reports (fifteen melanoma-related papers and books [33]) from which case descriptions and references. The word association strength (WAS) between the case title and case features (identified keywords) are combined within the word association profile called DePicT Profile Matrix [10]. The case structure (Fig. 1) comprises a case description and recommendation including image features, segmentation processes, identified keywords, and a word association profile. The DePicT Profile Matrix of melanoma has 260(5×52) elements for five cases as a melanoma stages and 52 identified keywords as features. The recommendation procedure of DePicT Melanoma CLASS is described in [10].

However, for image classification part, there are two classes of melanoma malignant and benign, which the recognition process comprises five main steps [10]:

- Sensor: The input data used for image processing comprises images and respective points of interest (POI). The image data should be in RGB format with a minimum resolution of 200 pixels, and images should include the skin spot to be examined. If there is more than one spot, the relevant spots must be marked as POIs.
- Feature generation: Features are developed to distinguish the spots based on their respective characteristics. The spots can then be divided into the categories malignant or benign.
- Feature Selection: After feature creation, the features are tested, with those that significantly improve detection selected and used while the rest are removed.
- Classification: Data input data using the selected features are categorized into the above two classes.
- System evaluation: After the system has been implemented, evaluation metrics are used to assess the performance of the system.

Region growing is a method for identifying similar areas on an image and then selecting them as a whole. As an initial step of this procedure, one or more seed points are selected. The color values of the neighboring pixels are then compared with those of the seed points; if they are sufficiently similar, the compared pixels are also selected and similarity comparisons are then performed with their neighbors. The algorithm terminates when there are no more sufficiently similar pixels. The image is then converted into a gray-value image as the first step. A 3×3 median filter (a modified decision-based unsymmetrical trimmed median filter) is used for noise reduction based on a received set of values—in this case, the gray values of the nine pixels—which are sorted, with the middle value selected as the new value for the current pixel. In the case of border pixels, missing values are filled with zeros. The brightness is adjusted to increase the contrast. The histogram of the gray values is rearranged to take advantage of the complete color space. Contrast of the gray values is readjusted to take advantage of the complete range of gray values. To segment the lesion region of interest (ROI), POIs are indicated within the relevant skin regions. To remove remaining smaller elements, a complementary image is formed and an opening process is used to close existing holes. Based on the characteristics of the skin spots occurring as results of melanoma, Table 1 presents twelve features considered in terms of the categories of color and shape [10].

**Table 1:** Image processing features

Category	Name	Inputs	Feature numbers
Color	Average RGB Channel	3	1-3
	Average HSV Channel	3	4-6
	Color Structure Descriptor	1	7
	Color Layout Descriptor	2	8-9
Shape	Principal Component Ratio	1	10
	Filled Fitted Ellipse	1	11
	Unfilled Fitted Ellipse	1	12

Because skin spots can be sharply differentiated or categorized by the color composition, the color values of the segmented images are considered and tested in both the RGB and HSV color spaces. Benign tumors tend to have much more circular or elliptical shape than malignant melanomas; accordingly, an ellipse is fitted around the ROI and the percentages of ROI pixels on the ellipse and outside of the ROI are used as features 11 and 12, respectively. To compare all twelve features, they are normalized (0,1) and stored in a feature vector for use in classification. This methodology is demonstrated in [10] by visualizing its steps as a process of selecting two 1600×900 pixel images from a dataset representing a benign and a malignant melanoma, respectively.

Thus, as it is explained in this section, users can fill their request based on their situation or insert their images from the affected area and our system can analyze their requested problem (i.e., text information and image), and recommends the solution related to that problem.

### DePicT Melanoma Deep-CLASS

Convolutional networks also known as CNNs, are a specialized kind of neural network for processing data. Its networks employ a mathematical operation called “convolution”. Convolution has three main characteristics that help and improve the machine learning system which are as follows [34]:

- Sparse interactions: this is accomplished by making the kernel smaller than the input and also computing the output requires fewer operations.
- Parameter sharing: more than one function in the model used the same parameter or a network has tied weights.
- Equivariant representations: the function is equivariant means if the input changes, the output changes in the same way.

The input and kernel is usually multidimensional array in machine learning applications which is created based on data and parameters respectively. Therefore, we use convolution over several axis at a time. For instance, if we have a two-dimensional image B as an input and we use a two-dimensional kernel K, our convolution after flipping the kernel to the input is as follows [34]:

1 $$ Conv(i,j) = (B*K)(i,j)= \Sigma_{i=1}^{n} \, \Sigma_{j=1}^{m} B(n,m)K(i-n, j-m)  $$

or in an equivalent way is:

2 $$  Conv(i,j) = \Sigma_{i=1}^{n} \, \Sigma_{j=1}^{m} B(i-n,j-m)K(n,m)  $$

and the function of the cross-correlation (which is without flipping) is as follows:

3 $$ Cross(i,j) = (B*K)(i,j)= \Sigma_{i=1}^{n} \, \Sigma_{j=1}^{m} B(i+n,j+m)K(n,m)  $$

**Fig. 2:**
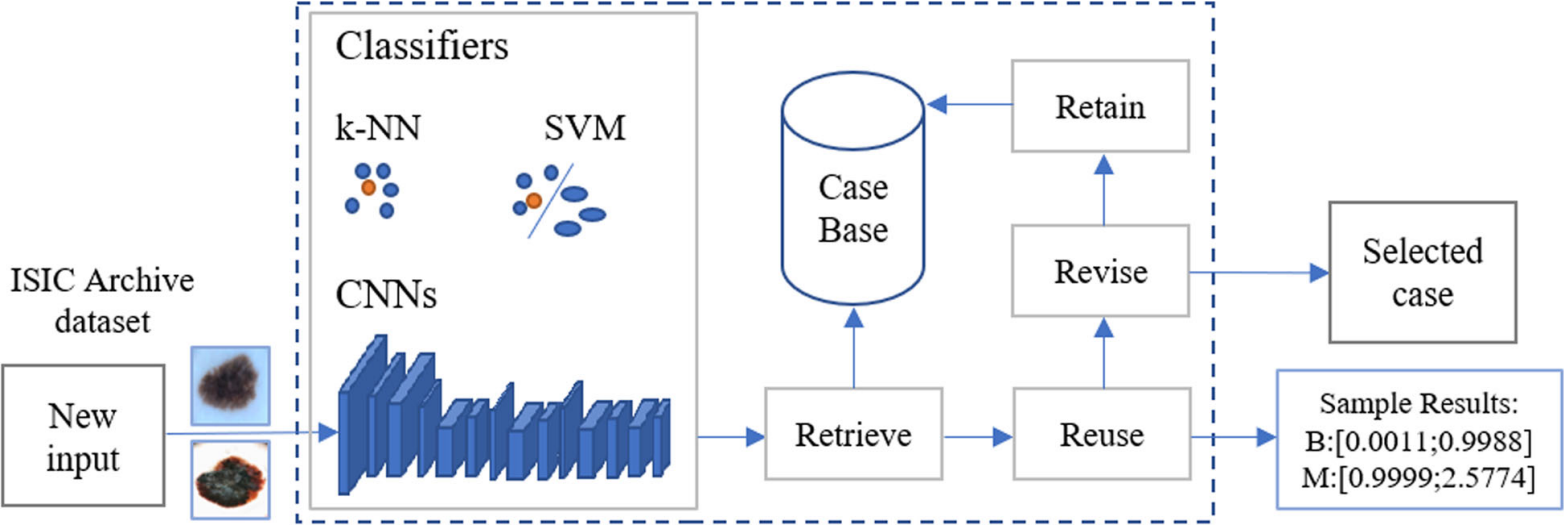
Overview of the proposed CBR system. DePicT Melanoma CLASS enriched with CNN method, adopted from [11]

In this study, by using CNN approach (Eqs. (1), (2)) considering 3-dimensional kernel, we applied an end-to-end CNN framework (machine learning system). As shown in Fig. 2, the goal of our proposed system called DePicT Melanoma Deep-CLASS is classifying new images to detect malignant melanoma using deep learning.

**Convolution** Convolution layer consists of several small trainable filters, typical filter sizes are ranging from 3*3*3 - 11*11*3 (the last corresponds to the 3 color channels of typical images) [34]. Each filter computes a dot product for each section of the input segment, by striding the filter. Important for this is the stride parameter, which indicates the amount of elements we slide the filter between two sets of calculations. At the border elements zero padding is required to fill in zeros for empty fields in the filter. Filters get sensitized regarding some visual features so that they activate when recognizing certain structural patterns. For example the first layer tend to learn low level features (e.g. edges). High-dimensional inputdata requires that not all neurons are fully connected with each other but instead only certain connections are established. Output dimensions are dependent of the height and width of the input and number of filters.

**Transposed Convolution (Deconvolution):** Transposed Convolution layer, also falsely labeled as Deconvolution, shares the same parameter with the Convolution layer (filter, stride, padding). It can be used to up sample data. Missing values are padded with zeros.

**HDF5 Input:** The HDF5 Input layer reads data from HDF5 files [35].

**Fig. 3:**
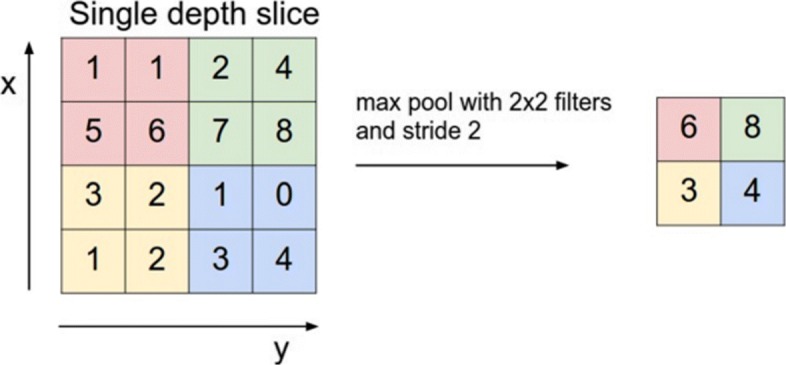
MAX pooling. forward propagation [36]

**Pool** In NN pooling layer is used to increase the computation performance and reduce the chance to over-fit by reducing the spatial information and therefore also the number of parameters [36]. Input of the layer is a blob with n * c * h_i * w_i dimensions, output is a blob with n * c * h_o * w_o dimensions. Kernel size specifies the layers kernel dimensions. Stride defines the number of elements the window slides over the blob. Pooling methods include ’MAX, AVE, and STOCHASTIC’. MAX pooling selects the numerical highest value of the given input Fig. 3. AVE pooling returns the average of given input. Stochastic pooling chooses via probability distribution samples of the activation. Larger activations have a higher chance to be chosen. Most common pooling is max pooling with a kernel size of 2x2, which results in a data reduction of 75% and outperforms other pooling methods in most cases [18].

**Crop** Crop layer has two blobs a input and one blob with the dimensions of the second blob as output [35]. Elements of the first blob get cropped so that they fit into the dimensions of the second blob. A parameter called axis defines the dimensions for cropping.

**Activation** Activation functions define the relation between Inputblob and Outputblob of a neuron [35]. For each element of the Inputblob one value is calculated for the output. In the following paragraphs a few functions will be introduced.

**Fig. 4:**
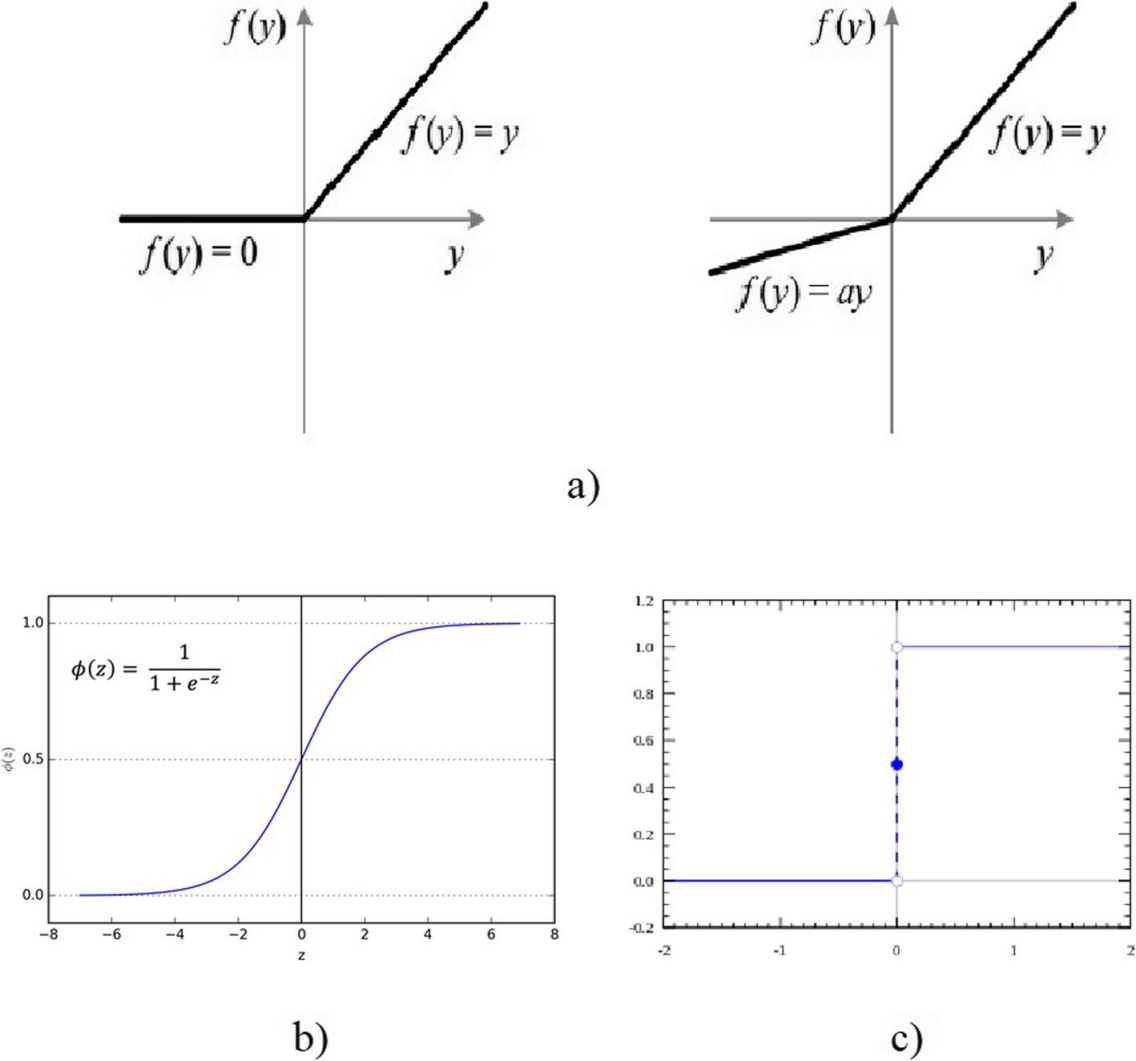
Different Activation Functions. **a** ReLU and Leaky ReLU [37], **b** Sigmoid Activation Function [37], **c** Step Activation Function [38]

- ReLU: ReLU functions receive one Inputblob and return one Outputblob of the same size. For every input value x the ReLU function returns x if x >0 and 0 if x ≤0. Leaky ReLUs as a special case multiply x with a if x ≤0, see Fig. 4a. ReLUs are efficient functions, they only need comparison, addition and multiplication, and profit from sparse activation. On the other hand they are non-differentiable at zero and can sometimes reach states in which they become inactive for essentially all inputs, which is also known as the ’Dying ReLU problem’.

- Sigmoid: Characteristically for Sigmoid functions is their ’S’-shaped form, also called Sigmoid curve. Shape and formula can be seen on Fig. 4b. Sigmoid function have a non-negative derivative at each point.

- Step Function: Step Function is a binary function, if input x exceeds a certain threshold the output is defined as 1, otherwise it is 0 Fig. 4c [38].

**Fully Connected Layer:** Fully Connected (FC) Layer is basically identical to multi-layer perceptron. This type of layer is very useful for classification and therefore often at the end of a network [39]. The number of neurons in the last layer then corresponds to the number of classes the network has to differentiate.

**Dropout** Dropout functions offer a solution for a serious problem of NN, over-fitting [12]. Randomly selected neurons (and also their connection) are deactivated during training based on a factor p. By doing so more than one neuron has to specialize on the same problem. Disrupting the network in this way leads to a more robust and redundant network.

**Flatten** Flatten reduce the dimensions of an Inputblob [35]. Input of the form n * c * h * w gets ’flattened’ to a vector of the form n * (c*h*w).

**Eltwise** Eltwise realize element wise operations for at least two Inputblobs of the same dimensions [35]. Common operations are sum, product and maximum. The resulting Outputblob has the same dimensions as the Inputblobs.

**Softmax** In classification tasks output is sometimes preferable in the form of a class probability. Therefore a Softmax layer is utilized to normalize input values and the sum of all outputs equals to one.

**Loss** Loss functions calculate the results of training by comparing the output with a ground truth [35]. Smaller loss is more preferable and indicates that the model is better at approximating the data. Results of loss are never negative. During the forward pass the loss is calculated, afterwards a separate backward pass to calculate the loss dependent gradient is required. Loss functions receive two Inputblobs, one with values in [0,1], indicating the probability $ \hat {p} $ for each of the *K* classes. The other blob holds labels *l*, representing the ground truth. In the following *N* represents the batchsize. There are three typed of loss that are i) Multinomial Logistic Loss, ii) Sum-of-Squares / Euclidean, and iii) Sigmoid Cross-Entropy Loss.

**Accuracy** Accuracy functions have a similar functionality as loss. Based on target labels they calculate accuracy values, but they require no backward step [35].

**Fig. 5:**
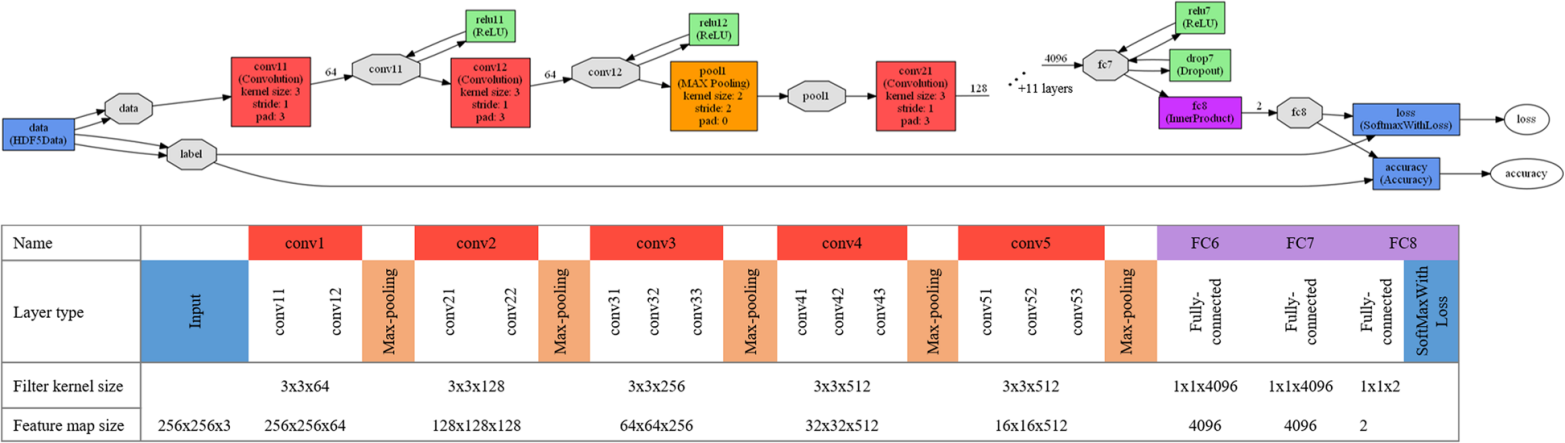
Overview of the CNN. Layer view and a MNIST digit classification example of a Caffe network - Sixteen layers excluding max-pooling [11]

**Fig. 6:**
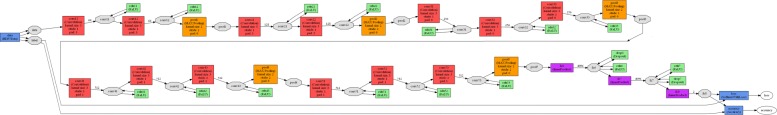
Complete layer view and a MNIST digit classification example of our Caffe network. Sixteen layers excluding max-pooling; convolution in red, max-pooling in orange and fully connected layers in violet

Therefore, CNNs are created of several convolutional layers (involving linear and nonlinear operators), pooling, inner products which are fully connected layers and softmax (combining with loss) and the architecture for its state-of-the-art has many parameters. Following the VGG 16 architecture [19], we built and fine tuned a 16 layermodel considering input data, input layer, last Fully Connected, and multinomial logistic loss. As shown in Fig. 5 (the complete net is also shown in Fig. 6), our model contains thirteen convolutional (series of conv layers: conv1-conv5) and three fully connected (FC) layers. The input image is the first layer (*h*×*w*×*d* which *h*×*w* is the pixel size and *d* is the color channel, here is 256×256×3). The configuration of our network is also illustrated at the bottom of Fig.5 regarding the filter kernel and feature map size represented by a three-dimensional array (*h*×*w*×*d* which *h* and *w* are spatial dimensions and d is the number of channels). Our requested problem is a two-way classification problems: malignant and benign classes, therefore, the last fully connected layer (FC8) has 2 channels which is the same as the number of classes (B and M, see also Fig.2 - Sample Results), while the first-two fully connected layers (FC6 and FC7) have 4096 channels. Therefore, the case representation is generated based on the features coming out of the last layer of our network. DePicT Melanoma Deep-CLASS used images from ISIC Archive dataset to train our classifier (CNN) and cases. After creation of the whole case base based on the labeled images, in retrieval process of new input image, a distance-based method can work over the case representation to find the matched class.

The process of classification started with the “Input” and finished with “Softmax” which are as follows:

- Input: as input images required in the format 256x256x3 and data has been rescaled to the desired format.
- Convolution: the second layer is “conv11” which is the convolution layer containing a kernel size of three, which corresponds to a filter size of 3x3. To prevent the decrease of spatial dimensions zero padding is applied. The number of 3x3 filter apllied equals 64 which is set by the number of output parameters. Further stride is set to 1 which applied for each pixel. The type of weight filler is defined based on [40], and the bias filler is set to constant value 0.2.
- ReLU: after the convolution layer, a layer of “relu11” which is a “Rectified-Linear” layer applied as an activation function. All other convolution layers are constructed as the first one, except that the number of output increases according to tabular [link to tabular with filterkernel size per layer]. Also each convolution layer is followed by a “ReLU”.
- Pooling: after every set of 2-3 convolution layers, a layer of “Pooling” is used to decrease the data size. A MAX function is used as pooling method. Kernel size and stride are set to 2. Therefore, for every 4 pixels from the input data, the highest value is chosen for the output data (effectively to reduce by half the number of elements in two dimensions.
- InnerProduct: following the last “Pooling” layer three Fully Connected Layers (FCL) as an “InnerProduct” are used. Input to those layers is treated as a simple vector and is matched on a number of output neurons organized in the form of a single vector with the size of 1 x number of output. The first two FCL possess 4096 output neurons, a weight filler of “gaussian” with a standardard deviation of 0.005 and a bias filler set to the constant value of 1. The third FCL possesses 2 output neurons, a weight filler of “gaussian” with a standardard deviation of 0.01 and a bias filler set to the constant value of 0. Only 2 output neurons are used for the third layer to match the number of different classes (benign and malignant) in the input labels of the neural network.
- Softmax: at the end of the network a layer of “Softmax” is applied as an output of our net.

## Datasets and tools

**Fig. 7:**
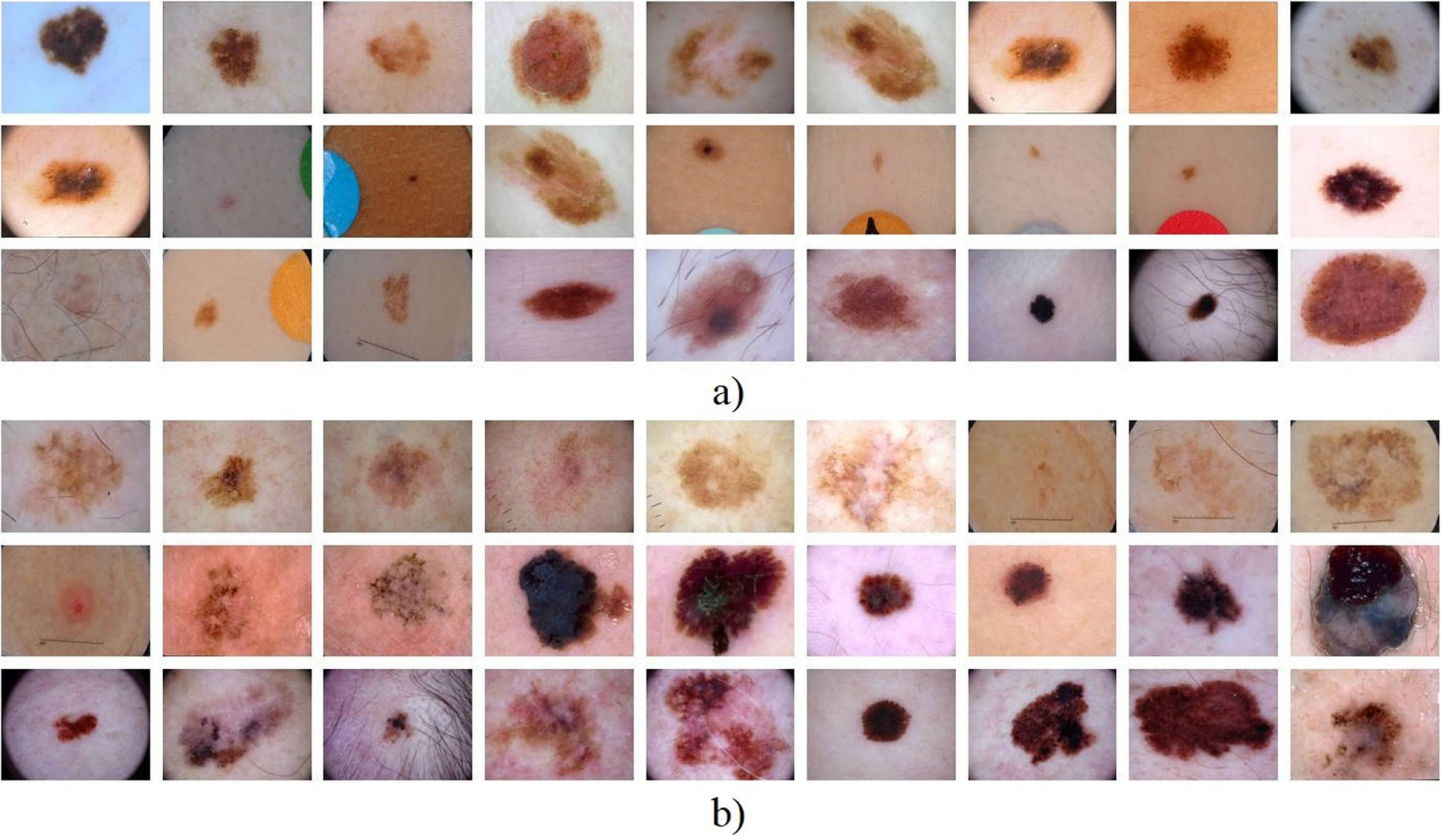
Sample images. Benign and malignant melanoma images from IS IC Archive dataset [22]. **a** B **b** MM

As it mentioned the ISIC Archive dataset [22] containing images of benign lesions and malignant melanoma was used for image-processing and classification (see Fig. 7). In the first round, 300 images for training and 100 for testing were utilized [10]. By continuing the previous study, we have first used this dataset and then increased the training and test images in the second round to 1796. A total of 1346 dermoscopy images comprising malignant and benign, were analyzed in this study (the second, third and fourth rounds) to train our classifiers and 450 images are used for these testings. Also, for comparing CNN with the previous classifiers, we have used CNN in the third and fourth tests following the first two tests.

As it is mentioned, CNNs consist of two major components; Convolution consists of several convolution and amongst other things pooling layers. The second component holds FC layers. Dropout layers separate those components to prevent overfitting. Often a softmax followed by a loss function concludes the network structure. However CNNS require many data for training. One way to handle this problem is data augmentation. Data Augmentation refers to the process of creating new data from existing datasets according to certain predefined rules [16]. It is useful when the existing amount of data is deemed as to small or homogeneous regarding some patterns. Classical augmentation techniques include: rotating, rescaling, flipping, translating, shearing and cropping the data.

For applying deep-learning, we have utilized Caffe [41] which is a deep learning framework developed by Berkeley AI Research (BAIR) [http://bair.berkeley.edu/]. Matlab (2017a) was utilized to develop DePicT Melanoma CLASS, in particular with the use of Image Processing Toolbox, Parallel Computing Toolbox, Matlab Compiler and Coder, and App Designer.

## Results and discussion

We evaluated the performance of the proposed system based on the evaluation scores (precision which is the repeatability, or reproducibility of the measurement results, sensitivity also known as recall is the probability of a positive test given that the patient has the disease, specificity is the probability of a negative test given that the patient is well, f-measure which is a is the harmonic mean of precision and recall and finally accuracy which is the proximity of measurement results to the true value), which are the most popular evaluation metrics and are defined by Eqs. (4)–(8), respectively. The evaluation scores of the methodologies for these applications were calculated as follows:

4 $$  Precision = \frac{TP}{TP+FP}  $$

5 $$ {\begin{aligned} Sensitivity &= Recall = \frac{number\, of\, correctly\, predicted\, malignant\, lesions}{number\, of\, malignant\, lesions} \\ & = \frac{TP}{TP+FN} \end{aligned}}  $$

6 $$ {\begin{aligned} Specificity &= \frac{number\, of\, correctly\, predicted\, benign\, lesions}{number\, of\, benign\, lesions} \\ &= \frac{TN}{FP+TN} \end{aligned}}  $$

7 $$ F-measure = 2 \times (Precision \times Recall) / (Precision + Recall)  $$

8 $$  Accuracy = \frac{TP+TN}{TP+TN+FP+FN}  $$

The measures are computed by utilizing the equation explained with the following conventions as follows:

- *TP* (True Positive): positive images classified as positive.
- *TN* (True Negative): Negative images classified as negative.
- *FP* (False Positive): Negative images classified as positive.
- *FN* (False Negative): Positive images classified as negative.

**Fig. 8:**
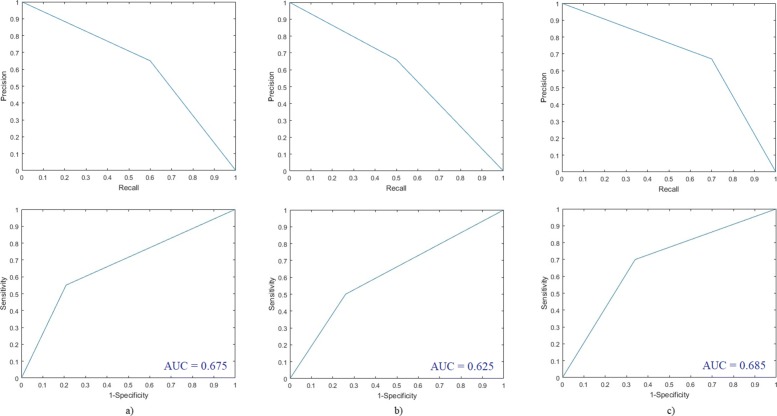
Evaluation results of DePicT Melanoma Deep-CLASS (CNN) in comparison with DePicT Melanoma CLASS. ROC and Precision-recall curves in the first and third round of training-testing: **a** k-NN, **b** SVM, and **c** CNN

**Fig. 9:**
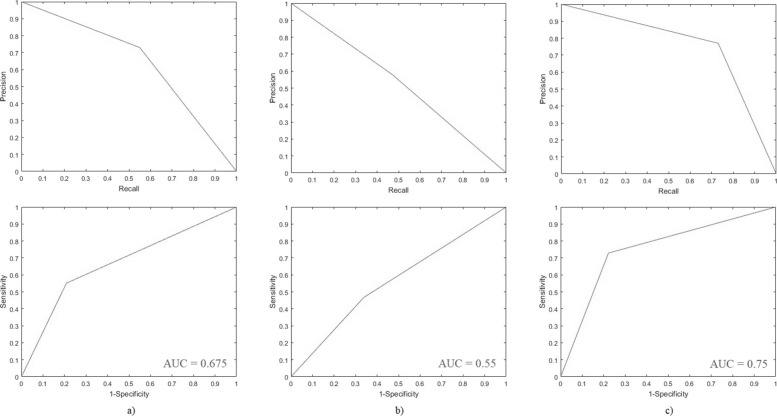
Evaluation results of DePicT Melanoma Deep-CLASS (CNN) in comparison with DePicT Melan oma CLASS. ROC and Precision-recall curves in the second and fourth round of trainingtesting: **a** k-NN, **b** SVM, and **c** CNN

DePicT Melanoma CLASS achieved appropriate results. Its performance in terms of comparison of its evaluation scores in these two rounds of testing is shown in Table 2. As shown in Fig. 8 (results for the first and third round of testing) and Fig. 9 (results for the second and fourth round of testing) based on the receiver operating characteristics (ROC), precision-recall and area under curve (AUC), DePicT Melanoma Deep-CLASS outperformed better.

**Table 2:** The comparison of evaluation scores (precision, recall (sensitivity), specificity, f-measure and accuracy) of DePicT Melanoma CLASS/Deep-CLASS

Classification and retrieval	TP	TN	FP	FN	Pre.	Rec.(Sen.)	Spec.	F-m.	Acc.
1st test: k-NN (300, 100)	30	34	16	20	0.65	0.6	0.68	0.62	0.64
1st test: SVM (300. 100)	25	37	13	25	0.66	0.5	0.74	0.57	0.62
2nd test: k-NN (1346, 450)	124	178	47	101	0.73	0.55	0.79	0.63	0.67
2nd test: SVM (1346. 450)	105	149	76	120	0.58	0.47	0.66	0.52	0.56
3rd test: CNN (300,100)	35	33	17	15	0.67	0.70	0.66	0.68	0.68
4th test: CNN (1346,450)	164	175	50	61	0.77	0.73	0.78	0.75	0.75

## Conclusions

Melanoma skin cancer is discussed as domain of current research in this paper. According to the available data on the incidence of malignant melanoma in Germany in the period from 1970 to 2012, there is an increase age-standardized incidence rates of melanoma from 3 cases to 19 cases per 100,000 inhabitants and year. Thus, the incidence of melanoma is over almost four decades has increased more than sixfold (633%) and every year more than 20,000 people suffer from malignant melanoma [42].

Therefore, early detection of melanoma is one of the key objectives in skin cancer treatment. The frameworks presented in this work play a significant role in attenuating inter- and intra-variability in medical and educational medical assistant systems. We proposed a case-based system for utilizing collected cases to support patients and healthcare providers through the early detection of melanoma. We used word association profiles obtained from requests in the form of text queries or filled-in questionnaires and both k-NN and SVMs to classify incoming images in our previous work and to develop DePicT Melanoma CLASS. In this study, we introduce DePicT Melanoma Deep-CLASS which is enriched by deep learning approach and CNN. A training and testing method were deployed for composing a comparatively accurate CNN model from sample images of dataset.

We have also compared these two case-based systems with the same images from the ISIC Archive dataset. Although further data analysis is necessary to improve its accuracy, CNN would be helpful for the early detection of malignant melanoma. Analysis of the results obtained by testing a melanoma dataset suggests that our enriched case-based system (via CNN which made case classification more efficient) for detecting malignant melanoma is fit for the purpose of supporting users by providing relevant information.

In the future, we will elaborate on DePicT Melanoma Deep-CLASS to make it more robust against noisy images by using normalization methods to improve the performance of our system and increase the accuracy. Also, further work will involve extending the training phase by using more images and more classes (e.g., different types and stages of melanoma skin cancers). In this regard, we are fine tuning our CNN to a 19-layer model (containing eleven convolutional (conv), five max-pooling and three fully connected (FC) layers) and our data is extracted from the “ISIC 2018: Skin Lesion Analysis Towards Melanoma Detection” grand challenge datasets [43, 44]. Additionally, the retrieval phase could also be further developed based on the new classes in regenerating a case representation.

## Data Availability

ISIC Archive dataset containing images of benign lesions and malignant melanoma was used in the current research.

## Abbreviations

AJCC: American joint committee on Cancer
AUC: area under curve
CBR: case-based reasoning
CNN: convolutional neural networks
DePicT Melanoma CLASS: detect and predict melanoma using case-based learning assistant system
DePicT: detect and predict diseases using image classification and text information
DL: deep learning
FC: fully connected
FCRN: fully convolutional residual networks
FN: false negative
FP: false positive
HSV: hue, saturation, and value
ISIC: international skin imaging collaboration
K-NN: k nearest neighbor
LLGMNN: log-linearized Gaussian mixture neural network
NCCN: national comprehensive cancer network
NN: neural network
POI: point of interest
RGB: red, green, and blue
ROC: receiver operating characteristics
ROI: region of interest
SVM: support vector machine
TN: true negative
TP: true positive
VGG: visual geometry group

## References

[1] Skin Cancer Facts & Statistics Melanoma. https://www.skincancer.org/skin-cancer-information/skin-cancer-facts/\#melanoma. Accessed 31 Oct 2018.

[2] Barnes B, Kraywinkel K, Nowossadeck E, Schönfeld I, Starker A, Wienecke A, Wolf U. Bericht zum krebsgeschehen in deutschland 2016: Robert Koch Institut; 2016, pp. 53–56.

[3] American Cancer Society (2017). Cancer Facts and Figures 2017. Genes Dev.

[4] Coit DG (2016). NCCN Guidelines Insights: Melanoma, Version 3.2016. J Natl Compr Cancer Netw JNCCN.

[5] Esteva A, Kuprel B, Novoa RA, Ko J, Swetter SM, Blau HM, Thrun S (2017). Dermatologist-level classification of skin cancer with deep neural networks. Nature.

[6] Mustafa S, Kimura A. A SVM-based diagnosis of melanoma using only useful image features. In: 2018 International Workshop on Advanced Image Technology (IWAIT). IEEE: 2018. 10.1109/iwait.2018.8369646.

[7] Zakeri A, Soukhtesaraie S. Automatic Diagnosis of Melanoma Using Log-Linearized Gaussian Mixture Network. In: 2017 24th National and 2nd International Iranian Conference on Biomedical Engineering (ICBME). IEEE: 2017. 10.1109/icbme.2017.8430224.

[8] Putten EV, Kambod A, Kambod M. Deep residual neural networks for automated Basal Cell Carcinoma detection. In: 2018 IEEE EMBS International Conference on Biomedical Health Informatics (BHI). IEEE: 2018. 10.1109/bhi.2018.8333437.

[9] Cherepkova O, Hardeberg JY. Enhancing dermoscopy images to improve melanoma detection. In: 2018 Colour and Visual Computing Symposium (CVCS). IEEE: 2018. 10.1109/cvcs.2018.8496604.

[10] Nasiri S, Jung M, Helsper J, Fathi M. Detect and Predict Melanoma Utilizing TCBR and Classification of Skin Lesions in a Learning Assistant System In: Rojas I, Ortuño F, editors. Bioinformatics and Biomedical Engineering. IWBBIO 2018. Lecture Notes in Computer Science, vol 10813. Springer International Publishing: 2018. p. 531–542.

[11] Nasiri S, Helsper J, Jung M, Fathi M. Enriching a CBR recommender system by classification of skin lesions using deep neural networks. In: Workshop Proceedings (CBRDL: Case-Based Reasoning and Deep Learning) of the 26th International Conference on Case-Based Reasoning (ICCBR 2018). Stockholm: 2018. p. 86–90.

[12] Srivastava N, Hinton G, Krizhevsky A, Sutskever I, Salakhutdinov J (2014). Dropout: A Simple Way to Prevent Neural Networks from Overfitting. J Mach Learn Res.

[13] Dahl GE, Sainath TN, Hinton GE. Improving deep neural networks for LVCSR using rectified linear units and dropout. In: 2013 IEEE International Conference on Acoustics, Speech and Signal Processing. IEEE: 2013. 10.1109/icassp.2013.6639346.

[14] Perez L, Wang J. The Effectiveness of Data Augmentation in Image Classification using Deep Learning. arXiv:1712.04621 [cs.CV]. 2017.

[15] Paulin M, Revaud J, Harchaoui Z, Perronnin F, Schmid C. Transformation Pursuit for Image Classification. In: 2014 IEEE Conference on Computer Vision and Pattern Recognition. Columbus, OH: 2014. p. 3646–53. 10.1109/CVPR.2014.466.

[16] Data Augmentation | How to use Deep Learning when you have Limited Data-Part 2. https: //medium.com/nanonets/how-to-use-deep-learning-when-you-have-limited-data-part-2-data-augmentation-c26971dc8ced . Accessed 23 Oct 2018.

[17] Krizhevsky A, Sutskever I, Hinton GE. ImageNet Classification with Deep Convolutional Neural Networks In: Pereira F, Burges CJC, Bottou L, Weinberger KQ, editors. Advances in Neural Information Processing Systems 25: 2012. p. 1097–105.

[18] Scherer D, Müller A, Behnke S. Evaluation of Pooling Operations in Convolutional Architectures for Object Recognition In: Diamantaras K, Duch W, Iliadis LS, editors. Artificial Neural Networks – ICANN 2010. Springer Berlin Heidelberg: 2010. p. 92–101. 10.1007/978-3-642-15825-4_10.

[19] Simonyan K, Zisserman A. Very Deep Convolutional Networks for Large-Scale Image Recognition. arXi v:1409.1556 [cs.CV]. 2014.

[20] Szegedy C, Liu W, Jia Y, Sermanet P, Reed SE, Anguelov D, Erhan D, Vanhoucke V, Rabinovich A. Going Deeper with Convolutions. arXiv:1409.4842 [cs.CV]. 2014.

[21] Yu C, Yang S, Kim W, Jung J, Chung KY (2018). Acral melanoma detection using a convolutional neural network for dermoscopy images. PLOS ONE.

[22] ISIC Archive. https://isic-archive.com/. Accessed 24 Oct 2018.

[23] Matsunaga K, Hamada A, Minagawa A, Koga H. Image Classification of Melanoma, Nevus and Seborrheic Keratosis by Deep Neural Network Ensemble. arXiv:1703.03108 [cs.CV]. 2017.

[24] He K, Zhang X, Ren S, Sun J. Deep Residual Learning for Image Recognition. CoRR: abs/1512.03385. 2015.

[25] González-Díaz I. Incorporating the Knowledge of Dermatologists to Convolutional Neural Networks for the Diagnosis of Skin Lesions. CoRR: abs/1703.01976. 2017. 10.1109/JBHI.2018.2806962 29994788

[26] Menegola A, Tavares J, Fornaciali M, Li LT, Fontes de Avila SE, Valle E. RECOD Titans at ISIC Challenge 2017. CoRR: abs/1703.04819. 2017.

[27] Szegedy C, Ioffe S, Vanhoucke V. Inception-v4, Inception-ResNet and the Impact of Residual Connections on Learning. arXiv:1602.07261 [cs.CV]. 2016.

[28] Li Y, Shen L. Skin Lesion Analysis Towards Melanoma Detection Using Deep Learning Network. CoRR: abs/1703.00577. 2017. 10.3390/s18020556 PMC5855504 29439500

[29] Sabouri P, GholamHosseini H, Larsson T, Collins J. A cascade classifier for diagnosis of melanoma in clinical images. In: Annual: International Conference of the IEEE Engineering in Medicine and Biology Society: 2014. p. 6748–51. 10.1109/EMBC.2014.6945177 25571545

[30] Kavitha JC, Suruliandi A, Nagarajan D, Nadu T (2017). Melanoma Detection in Dermoscopic Images using Global and Local Feature Extraction. Int J Multimed Ubiquit Eng.

[31] Sumithra R, Suhil M, Guru DS (2015). Segmentation and classification of skin lesions for disease diagnosis. Procedia Comput Sci.

[32] Nasiri S, Zenkert J, Fathi M (2017). Improving CBR adaptation for recommendation of associated references in a knowledge-based learning assistant system. Neurocomputing.

[33] Nasiri S (2018). Dynamic knowledge assets management to interactive problem solving and sustained learning - A collaborative CBR system in chronic and palliative care.

[34] Goodfellow I, Bengio Y, Courville A. Deep Learning. 2016: MIT Press.

[35] Caffe layers. Berkeley Artificial Intelligence Research. http://caffe.berkeleyvision.org/tutorial/layers.html. Accessed 31 Oct 2018.

[36] Pooling layer. http://cs231n.github.io/convolutional-networks/\#pool. Accessed 31 Oct. 2018.

[37] Activation functions: Neural networks. https://towardsdatascience.com/activation-functions-neural-networks-1cbd9f8d91d6. Accessed 31 Oct 2018.

[38] Sharma VA. Understanding activation functions in neural networks. https://medium.com/the-theory-of-everything/understanding-activation-functions-in-neural-networks-9491262884e0. Accessed 31 Oct 2018.

[39] Fully connected layers. Convolutional neural networks for visual recognition. http://cs231n.github.io/convolutional-networks/\#fc. Accessed 31 Oct 2018.

[40] Xavier Glorot X, Bengio Y (2010). Understanding the difficulty of training deep feedforward neural networks. Proc Thirteenth Int Conf Artif Intell Stat PMLR.

[41] Jia Y, Shelhamer E, Donahue J, Karayev S, Long J, Girshick R, Guadarrama S, Darrell T. Caffe: Convolutional Architecture for Fast Feature Embedding. arXiv preprint arXiv:1408.5093. 2014.

[42] Leitlinienprogramm Onkologie. S3-Leitlinie Melanom, Version 3.1, Juli. 2018. https://www.leitlinienprogramm-onkologie.de.

[43] Codella NCF, Gutman D, Celebi ME, Helba B, Marchetti MA, Dusza SW, Kalloo A, Liopyris K, Mishra N, Kittler H, Halpern A. Skin Lesion Analysis Toward Melanoma Detection: A Challenge at the 2017 International Symposium on Biomedical Imaging (ISBI), Hosted by the International Skin Imaging Collaboration (ISIC). arXiv:1710.05006. 2017.

[44] Tschandl P, Rosendahl C, Kittler H. The HAM10000 dataset, a large collection of multi-source dermatoscopic images of common pigmented skin lesions. 2018; Sci Data(5):180161. 10.1038/sdata.2018.161. 10.1038/sdata.2018.161 PMC6091241 30106392

